# Hope Buffers the Effect of Fear of COVID-19 on Depression among College Students: Insomnia as a Mediator

**DOI:** 10.3390/ijerph20043245

**Published:** 2023-02-13

**Authors:** Yingying Yao, Min Lin, Jianchao Ni, Jing Ni

**Affiliations:** 1 Counseling and Education Center, Xiamen University, Xiamen 361005, China; 2 Institute of Education, Xiamen University, Xiamen 361005, China; 3 School of Aerospace Engineering, Xiamen University, Xiamen 361005, China; 4 Faculty of Nursing, Jiujiang University, Jiujiang 332005, China

**Keywords:** fear of COVID-19, insomnia, depression, hope, college students

## Abstract

Background: In the period of the global pandemic, psychophysical problems induced by the fear of COVID-19 among college students deserve attention since the dormitory environment in college greatly increases the possibility of COVID-19 infection. Methods: A hypothesized mediated moderation model was to be verified using a cross-sectional study among 2453 college students. Fear of COVID-19, insomnia, hope, and depression were assessed by using the relevant scales. Results: (1) The fear of COVID-19 was positively correlated to depression (β = 0.365, t = 5.553, 95% CI = [0.236, 0.494]); (2) hope moderated the influence of the fear of COVID-19 on depression (β = −0.093, t = −4.066, 95% CI = [−0.137, −0.048]), as well as on insomnia (β = −0.095, t = −4.841, 95% CI = [−0.133, −0.056]); and (3) the mediated moderation model with hope as the moderator and insomnia as the full mediating variable between fear of COVID-19 and depression was verified (β = −0.060, 95% CI = [−0.093, −0.028]). Conclusions: The findings suggest that hope is a vital mechanism to explain the relationship between the fear of COVID-19 and depression in early adulthood. In practical application, mental health practitioners should focus on boosting hope and alleviating insomnia when addressing COVID-19-related depression issues among college students.

## 1. Introduction

At present, the global epidemic situation is still severe. Facts show that the number of confirmed cases and suspected cases is increasing, and media reports continue to report the latest situation of the epidemic. It is still unknown when the epidemic will end, meaning that people may coexist with the virus for a long time, bringing different levels of psychological stress and problems, such as tension, fear, and other stress reactions [1,2,3]. In April 2020, China entered the stage of normalized prevention and control, and under the context of that, regular teaching in colleges and universities has been gradually restored. Since colleges and universities are densely populated places and students live in a dormitory environment, the students’ fear of COVID-19, such as fear of a higher possibility of infection, might increase dramatically. Various studies have focused on COVID-19 fears during the pandemic [3,4]. However, limited attention was paid to college students [5]. How does the fear of COVID-19 influences students’ mental health? What can mental health practitioners do to minimize the influence? These were the key starting points of our study.

### 1.1. The Relationship between Fear of COVID-19 and Depression

Research shows that unforeseeable situations and the ensuing uncertainty might have detrimental effects on mental health [4]. COVID-19 can spread through social interactions and spread from person to person, causing various negative emotions and fears, leading to mental health issues such as depressive symptoms, e.g., sadness, attention deficit, altered appetite, restless sleep, and suicidal thoughts [6]. Liu et al. (2020) found that people are experiencing anxiety, depression, and other psychological abnormalities during the COVID-19 pandemic [7]. Ettman and his colleagues found that the morbidity of depressive symptoms among US adults was 24.7% prior to COVID-19 pandemic [8]. In contrast, during the pandemic, the prevalence was 52.5%. It was verified that the number of individuals showing depressive symptoms has increased with the COVID-19 pandemic [4,9,10], and the fear of COVID-19 accounted for unique variance in depression [11]. COVID-19 not only threatens an individual’s physical health, but also brings a huge psychological burden, which seriously affects individual mental health. In summary, this study proposes the following hypothesis:

**H1.**  *The fear of COVID-19 is related to depression.*

### 1.2. Hope Moderates the Relationship between Fear of COVID-19 and Depression

Although coronavirus diseases have a threatening influence, there is still space for positive psychology (such as hope) to relieve the expected ill effects on mental health by intensifying psychological resources [12,13,14,15]. Snyder (1989) carried out a systematic study on the positive psychological quality of “hope” and pointed out that hope means an internal motivation state, which is based on an interactively derived agency with goal-directed energy and pathways with planning to meet goals [16]. Hope is a persistent and specific goal-oriented process which may thwart the potential negative impact of stressors. Being able to set and adjust personal goals, constantly settle a matter, and strive to overcome obstacles to reach personal goals might help alleviate personal stress and decrease negative reactions [17]. A large body of research backs the idea that hope has been related to fewer adverse psychological outcomes. Hope was verified to alleviate adverse impacts on mental health [18] and reduce depressive symptoms by increasing motivation and adjusting coping styles [13]. Hopeful people have a more positive attitude toward life [13,19,20] and are more likely to recognize the positive aspects of life and view the development of the COVID-19 pandemic with a much more positive attitude [21,22]. Hope has been associated with depressive symptoms and less stress [23]. When faced with threats, individuals with higher hope will not be trapped in the current situation. Instead, they will be more likely to find directions for improvement from difficulties and fears and show firmer determination in pursuing goals [21]. Therefore, we proposed the following hypothesis:

**H2.**  *Hope moderates the relationship between the fear of COVID-19 and depression.*

### 1.3. Insomnia Mediates the Relationship between Fear of COVID-19 and Depression

Fear, as one of the common emotional reactions, should be paid attention to over time, and the physical effects of it should not be ignored [2]. Insomnia may be one of the possible physical outcomes of a negative emotional state, for the fear of COVID-19 showed a significant relationship with it [24]. Individuals in a state of high tension for a long time are prone to mental symptoms such as sleep disorders, resulting in varying degrees of mental disorders such as depression in severe cases. There is evidence that sleep quality may play a key role in depression, as well as anxiety [25,26]. There is a high incidence of sleep disorders in adults and children with anxiety and depression disorders [27]. Insomnia usually predates depression [28] and is associated with a two-fold increase in the incidence of depression compared with healthy sleepers [29]. Despite the fact that there is evidence that anxiety may predate sleep problems, studies on the directionality of sleep and anxiety are kind of mixed [30]. Fear of COVID-19 may lead to neurobiological alterations in the prefrontal–hypothalamus–amygdala and dopaminergic circuits, which is mediated at least in part by hormonal and neuropeptide alterations in the function of the hypothalamic–pituitary–adrenal axis [27,31]. These neuroendocrine changes might increase susceptibility to future stressors, sleep disorders, and mental health problems [32,33,34]. Insomnia is also increasingly conceptualized as a mechanism of the etiology of depression [29,35]. One meta-analytic study showed that insomnia is an important antecedent variable of depression and anxiety [36], and during the COVID-19 pandemic, anxiety and insomnia symptoms were common [37,38]. The stress related to the COVID-19 pandemic could affect depression directly and indirectly via insomnia [39]. In a national longitudinal study, insomnia was observed to play a mediating role between anxiety and depression [40]. Thus, on the basis of previous findings, we proposed the following hypothesis:

**H3.**  *Insomnia mediates the relationship between fear of COVID-19 and depressive symptoms.*

### 1.4. Hope Buffers the Relationship between Fear of COVID-19 and Insomnia

Hope is defined as the extent to which people have a positive belief in their future, being better able to cope with the negative impact of a traumatic event [41]. As mentioned above, hope is a vital coping process (hoping) and an important personality force (hopefulness) to buffer the negative effect of stressors on mental health. The discussion on the moderating role of hope between fear and insomnia is limited. Among the comorbid diseases of insomnia, ruminant thinking and anxiety are the most widely studied, and the conclusion made from the studies focused on the critical factor related to insomnia is meaningful for our study. One study [42] found that hope can cushion the negative impact of COVID-19 fears on rumination, which was proved to have a direct impact on insomnia [43,44]. Similarly, anxiety was found to be comorbid with insomnia symptoms, especially in individuals with depression, and hope with goal orientation and vivid pathway thinking helps to reduce anxiety and could be one of the important means of cognitive intervention, which has been proven to be effective in relieving insomnia [18,45]. Therefore, based on previous research, we proposed the following hypothesis:

**H4.**  *Hope buffers the effect of fear of COVID-19 on Insomnia.*

### 1.5. The Present Study

**Figure 1:**
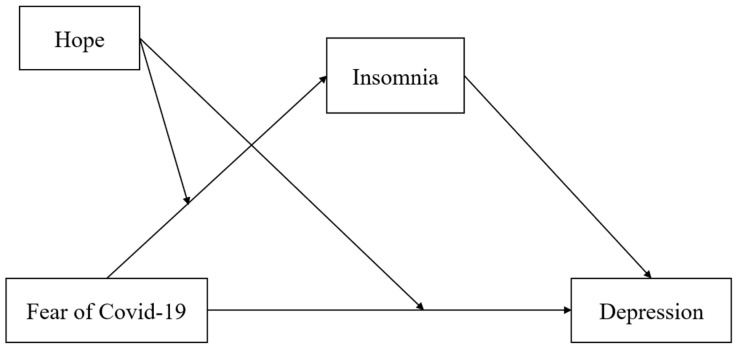
The mediated moderation model in the study.

Our study aimed to explore the relationship between the fear of COVID-19 and depression via insomnia and from the perspective of hope as an individual psychological force. A mediated moderation model is hypothesized in our study, as shown in Figure 1.

## 2. Materials and Methods

### 2.1. Participants and Sampling

This study conducted a cross-sectional design. An online survey was conducted among Chinese college students from October 2022 to December 2022. A mixed method of cluster sampling and convenience sampling was adopted to collect data by distributing links to questionnaires to Chinese college students. College students who have been diagnosed with schizophrenia were excluded. A total of 2453 participants completed the questionnaires, 52.5% of whom were female. The age ranged from 18 to 25 years old (Mage = 18.13; SD = 0.774).

All participants signed written consent forms about the purpose and procedure of our study online before accessing the questionnaire.

### 2.2. Measures

#### 2.2.1. Fear of COVID-19 Scale

The Fear of COVID-19 Scale (FCV-19S) [19] was used to measure the fear of COVID-19 among the general population. The FCV-19S consists of 7 one-dimensional items, using a five-point scale (from “1 strongly disagree” to “5 = strongly agree”). The total score is calculated by summing the score for each item, with the range from 7 to 35. Example items are “I cannot sleep because I’m worrying about getting COVID-19” and “My hands become clammy when I think about COVID-19”. The reliability was 0.883 in the study, according to Cronbach’s α.

#### 2.2.2. Athens Insomnia Scale

The degree of sleep deficiency of college students was recorded with the Athens Insomnia Scale [46]. The AIS includes eight questions, five of which evaluate the problem of sleep at night, and three evaluate the daytime consequences of insomnia. Each question is scored from 0 to 3 points, corresponding to “no problem at all” to “very serious problem”, respectively. A total score was summed, ranging from 0 to 24. The higher the score, the more serious the insomnia symptoms. Example items are “awakenings during the night” and “total sleep duration”. The cutoff for insomnia symptoms was 6. The internal consistency of AIS was acceptable (α = 0.800).

#### 2.2.3. Adult Dispositional Hope Scale

The 12-item Adult Dispositional Hope Scale [47], including two subscales (pathways and agency), was used to assess hope. The pathways subscale contains four items, which are accessed on a 7-point Likert scale (1-definitely false, 4-definitely true), and an example item is “I can think of many ways to get out of a jam”. Similarly, the agency subscale also contains four items, such as “I meet the goals that I set for myself”. The rest of the items are fillers and not calculated. The total score ranges from 8 to 32. The higher the score, the greater the hope. In this sample, the ADHS showed good internal consistency (*α* = 0.883).

#### 2.2.4. The Patient Health Questionnaire

The frequency of depressive symptoms over the past two weeks was assessed by the Patient Health Questionnaire (PHQ-9). It is a nine-item self-report measure on a 4-point scale ranging from 0 (none at all) to 3 (nearly every day). The total score of depressive symptoms was generated, ranging from 0 to 27, and a higher score indicates more severe depressive symptoms. In this study, the cutoff score of depressive symptoms was 5 [48], and the consistency was good (α = 0.911).

### 2.3. Data Analysis

A correlation analysis was used to test for bivariate correlations between the variables. Spearman correlations were used to analyze the correlation between variables since the normality assumptions were not fulfilled. Descriptive statistics on demographic characteristics and related variables were initially reported. Participants with and without depression were compared.

We used the PROCESS Model 8 to test a mediated moderation model with bootstrap confidence intervals for conditional effects. Moreover, a bias-corrected bootstrap procedure was used to generate 95% bias-corrected confidence intervals (CIs) from 5000 samples to test the significance of moderating mediating effects. SPSS 20.0 version was used for the above analyses. The two-tailed significance level was set at *p* < 0.05.

## 3. Results

### 3.1. Common Method Bias

We used Harman’s univariate analysis to examine common method biases [49] and conducted an exploratory factor analysis (EFA) included in all the measures mentioned above. Six factors with eigenvalues greater than 1 were founded, and they account for an acceptable percentage (26.00%) of the total variation compared to the criterion of 40%, meaning that there was no obvious common method bias in our study.

### 3.2. Descriptive Statistics

Students were divided into groups with or without depressive symptoms based on scores of PHQ-9. According to the original PHQ-9 development literature,1061 students who scored more than 5 on the PHQ-9 were assigned to the depressive group, accounting for 43.30% of all participants. Table 1 shows the *t*-test differences between the two groups. The results showed that the scores of the fear of COVID-19 and insomnia in the group with depressive symptoms were higher than those in the group without depressive symptoms (*t*-scores were -10.47 and -26.95, respectively; *p* < 0.001), and the scores of hope were lower in the group with depressive symptoms (*t* = 18.38; *p* < 0.001).

The descriptive statistics, as well as correlation coefficients between all the variables, are presented in Table 2. The fear of COVID-19 is significantly positively correlated with the insomnia score (*r* = 0.170, *p* < 0.01, and 95% CI = [0.128, 0.210]) and significantly positively correlated with depression (*r* = 0.246, *p* < 0.01, 95% and CI = [0.206, 0.286]). Insomnia is significantly positively correlated with depression (*r* = 0.601, *p* < 0.01, and 95% CI = [0.571, 0.629]). Hope has a negative correlation with both insomnia (*r* = −0.339, *p* < 0.001, and 95% CI = [−0.376, −0.301]) and depression (*r* = −0.458, *p* < 0.001, and 95% CI = [−0.490, −0.425]).

### 3.3. The Mediated Moderation Model Test

**Figure 2:**
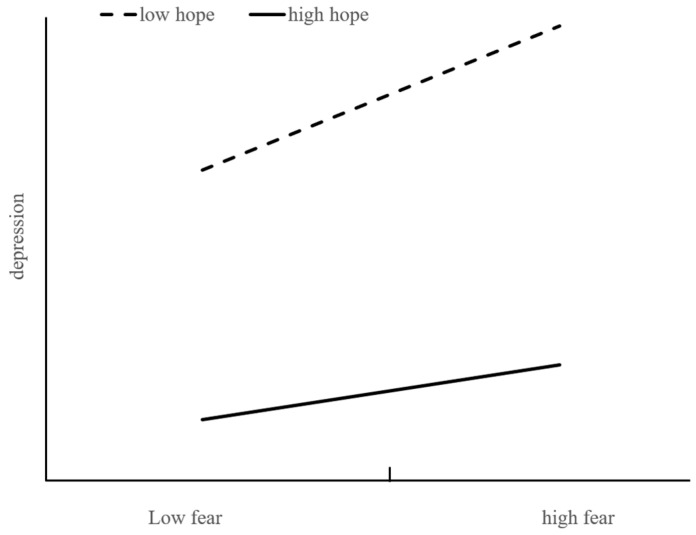
Hope as a moderator between fear of COVID-19 and depression.

Firstly, we used the path analysis to test the direct effect of fear of COVID-19 on depression and the hypothesized moderating role of hope on the direct path. SPSS PROCESS Macro (Hayes, 2013; Model 1) was adopted to investigate the moderation effect. For the significant correlations between demographic variables (gender and age) and psychosocial variables, they are controlled as the covariates in the following analysis. After controlling the covariates, fear of COVID-19 was found to be significantly related to depression (*β* = 0.365, *t* = 5.553, and 95% CI = [0.236, 0.494]), and the interaction between fear of COVID-19 and hope was significantly associated with depression; that means that hope moderated the relationship between fear of COVID-19 and depression significantly (*β* = −0.093, *t* = −4.066, and 95% CI = [−0.137, −0.048]) as presented in Table 3. Specifically, when the scores of hope were low (1 SD below), the effect of the fear of COVID-19 on depression was significantly positive (*β* = 0.150, *p* < 0.001, and 95% CI = [0.117, 0.184]). When the level of hope was high (1 SD above), the influence of the fear of COVID-19 on depression was also significantly positive but became weaker (*β* = 0.057, *p* = 0.001, and 95% CI = [0.024, 0.090]). Figure 2 illustrates the simple slopes of the fear of COVID-19 that predict depression for low hope (1 *SD* below) and high hope (1 *SD* above). Hypothesis 1 and Hypothesis 2 were supported.

**Figure 3:**
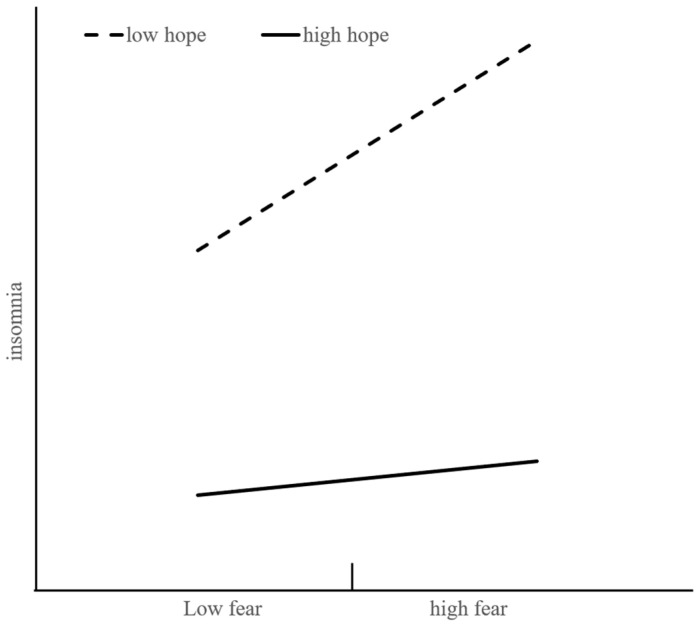
Hope as a moderator between fear of COVID-19 and insomnia.

Secondly, we used path analysis to test the effect of the fear of COVID-19 on insomnia and the hypothesis that hope acts as a moderator for the relationship. After controlling the gender and age as covariates, fear of COVID-19 was verified to be significantly related to insomnia (*β* = 0.118, *p* < 0.001, and 95% CI = [0.080, 0.156]), and the interaction of fear of COVID-19 and hope was significantly associated with insomnia, meaning that hope moderated the positive relationship between fear of COVID-19 and insomnia significantly (*β* = −0.095, *t* = −4.841, and 95% CI = [−0.133, −0.056]). Specifically, when the scores hope were low (1 *SD* below), the effect of fear of COVID-19 on insomnia was significantly positive (*β* = 0.113, *p* < 0.001, and 95% CI = [0.151, 0.255]). When the level of hope was high (1 SD above), the effect of the fear of COVID-19 on depression was also not significant (*β* = 0.018, *p* = 0.205, and 95% CI = −0.018, 0.084]). Figure 3 illustrates the simple slopes of the fear of COVID-19 that predict insomnia for low hope (1 SD below) and high hope (1 SD above). Thus, Hypothesis 2 was supported.

Thirdly, the SPSS PROCESS Macro (Hayes, 2013; Model 8) was used to investigate the mediated moderation effect. The results can be read form the table three. Fear of COVID-19 was positively related to depression (*β* = 0.154, *t* = 2.769, and 95% CI = [0.045, 0.263]), and insomnia was positively related to depression (*β* = 0.635, *t* = 32.067, and 95% CI = [0.596, 0.674]). Meanwhile, the interaction of fear of COVID-19 and hope was not related to depression (*β* = −0.033, *t* = −1.699, 95% CI = [−0.070, 0.005]). This means that the hypothesized mediated moderation model was validated (*β* = −0.060, 95% CI = [−0.093, −0.028]), and the moderating effect of hope was completely mediated by insomnia. The mediating effect of insomnia was 0.042, 95% CI = [0.027, 0.057]. The confidence interval of the mediating effect does not contain zero, meaning that the mediating effect is significant in our study. Hypothesis 3 and Hypothesis 4 were verified.

## 4. Discussion

The authors should discuss the results and how they can be interpreted from the perspective of previous studies and of the working hypotheses. The findings and their implications should be discussed in the broadest context possible. Future research directions may also be highlighted.

### 4.1. Significance of the Study

Our study focused on the influence of the fear of COVID-19 on depression among college students during the COVID-19 pandemic and explored the potential mechanism of the moderating effect of hope and mediating role of insomnia. This study extends the literature concerning COVID-19 by highlighting the role of psychological structure (hope), while also responding to calls from academics to explore factors that can prevent the fear of COVID-19 [50]. Firstly, it verified the moderating role of hope, which buffers the effect of the fear of COVID-19 on depression and the impact on insomnia. Hope, as an important positive character, has only received limited attention since the outbreak of the pandemic [50,51]. The study enriches the research on the hope theory under the background of the COVID-19 pandemic and expands a meaningful attempt to focus on the positive psychological structure. It also promotes the exploration and enlightenment of mental health care for college students from the perspective of hope. Secondly, it verified the mediated moderation model with insomnia as the full mediation variable through which hope alleviates the effect of the fear of COVID-19 on depression. As far as we know, this is the first study to investigate the mediating role of insomnia in the relationship between the fear of COVID-19 and depression. The findings deepen our understanding of the positive role of hope on the physical state, as well as on the mental state.

### 4.2. Fear of COVID-19 and Depression

The idea that the fear of COVID-19 has a significant impact on depression was verified in our study. The higher the fear of COVID-19, the more severe the depressive symptoms. In our studies, the detection rate of depressive symptoms among college students in the past two weeks was 43.30%, which is much higher than that based on community [52]. COVID-19 risk is a specific risk trigger similar to other physical diseases, which will induce a “fight or flight” reaction [53]. The unpredictable situation and uncertainty brought about by COVID-19 have adverse effects on mental health [4]. Risk perception and its related factors were found to significantly influence the mental health of public-health-crisis populations. Emotional risk perception is positively correlated with depression, while distance perception, cognitive risk perception, prevention, and control policy support are negatively correlated with depression [54]. The dormitory environment in the university has a high risk of COVID-19 infection, which may generate the fear of COVID-19 and be directly reflected in the mental health state in the form of, for example, anxiety or depression [4,50,55] among college students. A long period of possible contamination and social distancing sharply increases the individual’s feelings of uncertainty and fear of being infected; meanwhile, it steeply reduces interpersonal contacts and interpersonal support, finally increasing the possibility of mental health problems.

### 4.3. Hope as a Moderator on the Relationship between Fear of COVID-19 and Depression

Psychological strength, such as hope, plays an indispensable role in struggling with the pandemic. Hope was coded as one of the most frequent positive emotional connotations in a qualitative study [53]. Our study confirms hope as a moderator on the relationship between COVID-19 fear and depression. As a personal goal-based belief, hope acts as a buffering factor against affective disorders [51,56]. One characteristic of people with depression is that they find it difficult to make decisions, and a high level of hope facilitates more realistic goals and the motivation to deal with stressors, along with the awareness of pathways and the self-confidence and pathways to achieve goals [47]. Hope also protects people from depression, for a hopeful individual would make adaptive interpretations of adverse surroundings, see them as being ephemeral and understanding that they can be replaced by better situations [57]. Moreover, hopeful individuals probably make adaptive adjustments influencing both their appraisal of and method of coping with the stressors confronted by them [58].

### 4.4. Discussion on the Mediated Moderation Model

The study verified the moderating role of hope on the relationship between fear of COVID-19 and depression through the full mediation of insomnia. The COVID-19 pandemic has always been an important source of personal pressure [4], and the stress experienced by persons with fear triggers anxiety [59], which is one of the predisposing factors of insomnia and sharply reduces one’s sleep quality. Before COVID-19, the prevalence of insomnia was 4–15% [60,61], and during COVID-19 [62] and in our study (63.8%), insomnia is much more highly prevalent. Insomnia plays a significant role in the development and maintenance of depressive symptoms and is one of the best intervenable risk factors for depression [61]. Moreover, the interventions targeting insomnia have been proven to prevent the development and worsening of depressive symptoms [61]. Hope is also verified to cushion the relationship between the fear of COVID-19 and insomnia. Robust evidence demonstrates that cognitive behavioral therapy is effective in managing acute insomnia symptoms [18,45]. Lindsay and his colleagues have found that the external locus of control is a psychological construct linked to insomnia [63]. Individuals with a high level of hope may move from the external locus of control to the internal–external locus of control to encourage them to think about the goal and the corresponding pathway and then increase their control. The complete mediation of insomnia between the fear of COVID-19 and depression reminds us that attention to students’ sleep quality should also be an essential entry point for university mental health services.

### 4.5. Implications for Practice

The findings in our study are consistent with the recommendation of the World Health Organization that hope should be considered as one of the critical elements in psychological first-aid intervention used in humanitarian crises and complex emergencies globally.

In terms of application, the observations in the present study imply that mental health practitioners would be focusing on an individual positive psychological structure for college students when conducting mental health interventions during the COVID-19 pandemic. Mental health practitioners are advised to design interventions to minimize the adverse influence of COVID-19 both on an individual and collective level. At the individual level, practitioners can help college students to clarify the pressure they are facing, shape sufficiently clear goals, and figure out the feasible pathway to achieve their goals based on the evidence-informed interventions of the hope theory. At the collective level, group therapy focused on hope arousal [64] was found to be useful in elevating the level of hope and cultivating students’ positive psychological quality. Taking advantage of the positive role of hope to cope with depression constitutes a feasible peer-to-peer, group-based program that is well-received by its target group [65].

## 5. Limitations and Implications for Future Research

Several limitations of our study are worth noting. Firstly, the cross-sectional design does not allow us to establish temporal priorities among target variables or to make causal inferences. Empirical evidence showed that a higher fear of COVID-19 is related to mental health problems [66], and some others showed the direction from COVID-19-related emotion to fear of COVID-19 [55,67]. In our study, we assumed that the fear of COVID-19 exacerbates insomnia, which in turn exacerbates depression. However, longitudinal studies are still called for to further examine the causal relationship between variables in the future. Secondly, because of the characteristics of the sample, there may be some restrictions on the promotion of the findings. All the participants are college students with ages ranging from 18 to 25, and they should be more cautious about whether the results are applicable to other groups. For example, for the elderly, there are significant differences in insomnia, such more physiological causes. Thirdly, the pandemic prevention and control measures in the university are much stricter, for the environment in university is clustering, but it is unknown whether this stricter pandemic management mode increases students’ fear of COVID-19 or increases students’ sense of security to reduce their fear. Therefore, the antecedents of fear of COVID-19 are also worthy of further study in the future.

## 6. Conclusions

The findings imply that hope is a vital mechanism to explain the relationship between the fear of COVID-19 and depression among college students, which depends on the levels of hope and is fully mediated by insomnia. When addressing COVID-19 related mental health issues, especially the preventions and interventions for depression could focus on elevating hope and alleviating insomnia. It is suggested that mental health practitioners in colleges and universities need to pay full attention to both the physical state and physiological state induced by the fear of COVID-19 among college students. Mental health practitioners are advised to design the depression interventions from the perspective of hope because of its buffering effect in reducing the adverse influence of COVID-19 fear both on physical and mental health.

## Figures and Tables

**Table 1:** Results of *t*-test of concerned variables between the groups with and without depressive symptoms.

Variables	With Depressive Symptoms (*n* = 1061)	Without Depressive Symptoms (*n* = 1392)	*t*
Fear of COVID-19	17.19 ±6.22	14.62 ± 5.85	−10.47 ***
Insomnia	7.06 ± 3.48	3.64 ± 2.56	−26.95 ***
Hope	2.62 ± 0.46	2.97 ± 0.48	18.38 ***

Note: The cutoff score for with and without depressive symptoms is 5 in this study. *** *p* < 0.001.

**Table 2:** Descriptive statistics and correlations between variables.

	M ± SD	1	2	3	4	5	6
1 Gender	——	——					
2 Age	18.13 ± 0.774	−0.050 *	——				
3 Fear of COVID-19	15.73 ± 6.14	0.155 **	0.000	***0.883***			
4 Insomnia	5.12 ± 3.44	0.068 **	0.001	0.170 **	***0.800***		
5 Hope	2.82 ± 0.50	−0.024	0.045 *	−0.153 **	−0.339 **	***0.883***	
6 Depression	4.61 ±4.22	0.075 **	−0.012	0.246 **	0.601 **	−0.458 **	***0.875***

Note: The bold and italic numbers on the diagonal represent the internal consistency coefficient of the sales. The gender variable was coded as 1 = male, and 2 = female; * *p* < 0.05, and ** *p* < 0.01.

**Table 3:** The results of mediated moderation model test.

	Depression (First Step)			Insomnia (Second Step)			Depression (Third Step)		
	*β*	*t*	95% CI	*β*	*T*	95% CI	*β*	*t*	95% CI
Gender	0.240	1.562	[−0.061, 0.540]	0.284	2.160	[0.026, 0.541]	0.059	0.461	[−0.193, 0.312]
Age	0.042	0.429	[−0.150, 0.234]	0.111	1.319	[−0.054, 0.275]	−0.028	−0.344	[−0.190, 0.133]
Fear of COVID-19	0.365	5.553	[0.236, 0.494]	0.333	5.906	[0.222, 0.443]	0.154	2.769	[0.045, 0.263]
Hope	−2.288	−6.296	[−3.001, −1.576]	−0.707	−2.272	[−1.318, −0.097]	−1.839	−6.023	[−2.438, −1.240]
Insomnia							0.635	32.067	[0.596, 0.674]
Fear of COVID-19 *Hope	−0.093	−4.066	[−0.137, −0.048]	−0.095	−4.841	[−0.133, −0.056]	−0.033	−1.699	[−0.070, 0.005]
R^2^	0.233			0.130			0.460		
F	148.544			72.995			347.130		

Note: Fear of COVID-19 *Hope: the interaction of Fear of COVID-19 and Hope.

## Data Availability

The data that support the findings of this study are available upon request from the author YY. The data are not publicly available due to their containing information that could compromise the privacy of research participants.
